# The many faces of parasite calreticulin

**DOI:** 10.3389/fimmu.2023.1101390

**Published:** 2023-03-10

**Authors:** Diego Esperante, Ana Flisser, Fela Mendlovic

**Affiliations:** ^1^ Plan de Estudios Combinados en Medicina (PECEM), Facultad de Medicine, Universidad Nacional Autonóma de México (UNAM), Mexico City, Mexico; ^2^ Departamento de Microbiología y Parasitología, Facultad de Medicina, Universidad Nacional Autonóma de México (UNAM), Mexico City, Mexico; ^3^ Facultad de Ciencias de la Salud, Universidad Anahuac Mexico Norte, Huixquilucan, Mexico

**Keywords:** calreticulin, immune response, complement inflammatory disorders, vaccine, cancer

## Abstract

Calreticulin from parasites and its vertebrate hosts share ~50% identity and many of its functions are equally conserved. However, the existing amino acid differences can affect its biological performance. Calreticulin plays an important role in Ca^2+^ homeostasis and as a chaperone involved in the correct folding of proteins within the endoplasmic reticulum. Outside the endoplasmic reticulum, calreticulin is involved in several immunological functions such as complement inhibition, enhancement of efferocytosis, and immune upregulation or inhibition. Several parasite calreticulins have been shown to limit immune responses and promote infectivity, while others are strong immunogens and have been used for the development of potential vaccines that limit parasite growth. Furthermore, calreticulin is essential in the dialogue between parasites and hosts, inducing Th1, Th2 or regulatory responses in a species-specific manner. In addition, calreticulin participates as initiator of endoplasmic reticulum stress in tumor cells and promotion of immunogenic cell death and removal by macrophages. Direct anti-tumoral activity has also been reported. The highly immunogenic and pleiotropic nature of parasite calreticulins, either as positive or negative regulators of the immune response, render these proteins as valuable tools to modulate immunopathologies and autoimmune disorders, as well as a potential treatment of neoplasms. Moreover, the disparities in the amino acid composition of parasite calreticulins might provide subtle variations in the mechanisms of action that could provide advantages as therapeutic tools. Here, we review the immunological roles of parasite calreticulins and discuss possible beneficial applications.

## 1 Introduction

Parasites have coevolved with their mammalian hosts during eons, thriving in a hostile microenvironment characterized by a robust immune response. Protozoa and helminths, as well as ectoparasites have adapted and developed strategies to regulate the host’s immune response through the production and secretion of immunomodulatory molecules (1–3). These strategies permit chronic infection, continuation of their life cycle and transmission; and include molecules that regulate innate and adaptive immune cells, as well as soluble mediators (2, 4, 5). The constant interplay between parasites and their hosts has shaped the way the immune system functions and self-controls to minimize damage. The therapeutic use of helminth infections or their products as immunomodulators could help modify the overactive response seen in inflammatory and autoimmune disorders.

Calreticulin (termed Calr or CRT) is a multifunctional conserved protein and has been identified in different parasites, including protozoa, ectoparasites and helminths. The structure, as well as the functions of Calr are well preserved through evolution. It was first identified as an endoplasmic reticulum (ER)-residing protein but it is now well established as an intracytoplasmic and extracellular membrane component of most eukaryotic cells, as well as a secreted molecule in the extracellular milieu (6, 7). Parasite Calr is a pleiotropic molecule that participates in fecundity, infectivity, and modulation of the host immune response. Anti-angiogenic and anti-tumor properties are also one of the many facets of Calr. The aforementioned characteristics are shared by Calr of many studied species (8–11). Still, subtle species-specific differences have been described, as shown in (**Table 1**).

**Table 1:** Functions and immune response elicited by parasite calreticulin.

Parasite	Type of Calr	Experimental Model	Calr functions	Immune response	Reference
*Trypanosoma cruzi*	Recombinant	*In vitro* (EAhy926 endothelial cells)	Antiagiogenesis, tumor growth inhibition	Increase in tumor immunogenicity	Abello-Cáceres et al. (12)
*Trypanosoma cruzi*	Recombinant (fragments)	*In vitro* (human skin fibroblasts)	Cutaneous wound repair, fibroblast migration	–	Ignacio Arias et al., (13)
*Trypanosoma cruzi*	Native	*In vivo* (murine)	Increased parasite infectivity, binding of C1q and antibodies	Antibody synthesis; promotion of parasite phagocytosis by macrophages; inhibition of classical complement pathway	Ramírez et al. (14)
*Trypanosoma congolense*	Recombinant	*In vivo* (murine)	Prolonged survival in immunized mice; decrease parasitemia	Specific IgG production	Bossard et al. (15)
*Entamoeba histolytica*	Native	*In vitro* (peripheral blood mononuclear cells)	Modulation of immune response	Mixed Th1-Th2 response in different disease stages	González-Rivas et al. (16)
*Taenia solium*	Recombinant	*In vivo* (hamster)	Reduced parasite load in immunized hamsters, goblet cell hypertrophy	Production of IL-4, IL-10 (Th2) and IFN-g (Th1); isotype switch to IgA	León-Cabrera et al. (17)
*Taenia solium*	Recombinant and parasite extracts	*In vivo* (hamster)	Induction of immune response	Th2 response cytokines production (IL-4, IL-5, IL-10); synthesis of IgG	Mendlovic et al. (5)
*Taenia solium*	Recombinant	*In vitro* (cancer cell lines: HeLa, MCF7, SW480, PC3, MDA-MB-231, SKOV3)	Reduction of proliferation and cellular viability of tumor line cells, inhibition of cell-cell interactions	Binding of SRF on cancer cells leading to immunomodulatory effect	Schcolnick-Cabrera et al. (11)
*Heligmosomoides polygyrus*	Recombinant	*In vitro* (spleen and mesenteric lymph node cells)	Th2 immune response activation	Production of Th2 response cytokines (IL-4, IL-10); synthesis of IgG; binding of SR-A on DCs	Rzepecka et al. (18)
*Brugia malayi*	Recombinant	*In vitro* (splenocytes, mesenteric lymph nodes, splenic and peritoneal macrophages) and *in vivo* (murine)	Immunization with protein reduced parasite burden, immunogenic effect	Production of Th1 response cytokines (TNF-a, IL-6, IL-12, IFN-gamma) and, to a lesser extent Th2 (IL-2, IL-10); induction of humoral memory response; activation of macrophages	Yadav et al. (19)
*Trichinella spiralis*	Recombinant	*In vivo* (murine)	Binding of C1q, reduced immune response against parasitic larvae	Inhibition of classical complement pathway; reduced phagocytic activity of macrophages	Zhao et al. (20)
*Schistosoma japonicum*	Recombinant	*In vitro* (mouse dendritic cells and splenocytes)	Immune response against parasite schistosomula, maturation of DCs	Stimulation of DCs; production of TNF-a, IFN-gamma and IL-4; lymphocyte proliferation; Th1 response-skewing	Ma et al. (21)

The focus of this review is to highlight the diverse functions of parasite Calr as a strategy to evade the immune response and as a potential tool to treat immune mediated diseases, develop anti-parasite vaccines or to devise novel anti-neoplastic compounds.

## 2 Calreticulin: Structure, properties and functions

Mammalian Calr is a highly conserved 46kDa protein involved in many essential cellular functions. Two Calr genes have been identified in mammals, the Calr2 isoform is only expressed in testis. In parasites, the gene that encodes for Calr exists as a single gene that displays interspecies sequence variability (22). The level of expression of Calr varies according to the developmental stage of a particular parasite, as well as the parasite’s level of activity (including infection). (23, 24). Calr was first discovered as a luminal Ca^2+^ binding protein controlling intracellular Ca^2+^ homeostasis and as a chaperone involved in the proper folding of N-linked glycoproteins in the ER. It contains a signal peptide for secretion that is cleaved in the mature protein and directs it to the ER and secretory pathway, as well as a C-terminal tetrapeptide KDEL (Lys, Asp, Glu and Leu) that serves as an ER retention signal (25–27).

Calr is expressed in all nucleated cells except yeasts. The functions of Calr go beyond cellular Ca^2+^ homeostasis and quality control of synthesized glycoproteins, both associated with Calr residing in the ER. Although it was first localized to the ER, Calr has since been observed in the nucleus, cytoplasm, and cell membrane, as well as in the extracellular space and thus, has been shown to participate in many functions including, gene expression, cell adhesion, migration, wound healing, cancer, efferocytosis and immune modulation (6, 28).

Calr contains 3 highly conserved domains: a globular N domain (residues 18-186), an intermediate P domain that contains 2 sets of 3 highly conserved repeats (residues 187-286) and a C domain that includes a short hydrophobic stretch (residues 287-310) but is mainly acidic and binds Ca^2+^ with high capacity and low affinity, contributing to ~50% of the Ca^2+^ storage and buffering within the ER (residues 287-417) (26).

X-ray analysis of human (Hs) and mouse (Mm) Calr showed that the N domain together with residues 303-366 of the C domain comprise the globular domain with an antiparallel sandwich structure composed of 8 β strands. The C-terminal of the C domain collaborates with 2 central β strands and a long a helix. The globular domain binds Zn^2+^ ions and contains a carbohydrate binding site, as well as a high affinity Ca^2+^ binding site (29, 30). The N-/C-globular domain also has a conserved peptide-binding site that lies at the edge of the lectin site (30). In addition, circular dichroism (CD) experiments show that the globular domain is the Ca^2+^ and Zn^2+^ responsive unit (31).

NMR studies show that the intermediate P domain forms a hairpin structure stabilized by 3 short antiparallel β sheets that allow the proximity of the N and C domains and a helical turn at the tip of the hairpin (32). The tip is well structured but the 2 ends that connect with the C-terminal of the N domain and the N-terminal of the C domain are disordered (PDB ID: 1K9C; 33). The flexibility of Calr involves oscillations of the P domain and free C domain (34).The P domain contains two sets of 3 repeats each composed of proline-rich repeat sequences, a 17 amino acid, type 1 motif and a 14 amino acids type 2 motifs that may bind to oligosaccharides (32, 33, 35). The proline-rich P domain is also involved in the interaction with other chaperones and foldases within the ER (36, 37). However, different analyses show contrasting results regarding the Ca^2+^ binding capacity of the P domain. *In vitro* studies suggests that this region also binds Ca^2+^ with high affinity (38, 39), while CD experiments suggest that metal ions do not induce structural rearrangements of the P domain. Nevertheless, the P domain shows high conformational flexibility (31).

The acidic C domain involved in Ca^2+^ buffering in the ER binds this cation with high capacity and low affinity and has a disordered structure that transitions to an ordered conformation in the presence of physiological Ca^2+^ variations in the ER (40). It is currently unknown if the C domain folds independently of the globular domain. Still, cellular Ca^2+^ concentrations impact Calr functions and subcellular localization (40, 41).

**Figure 1:**
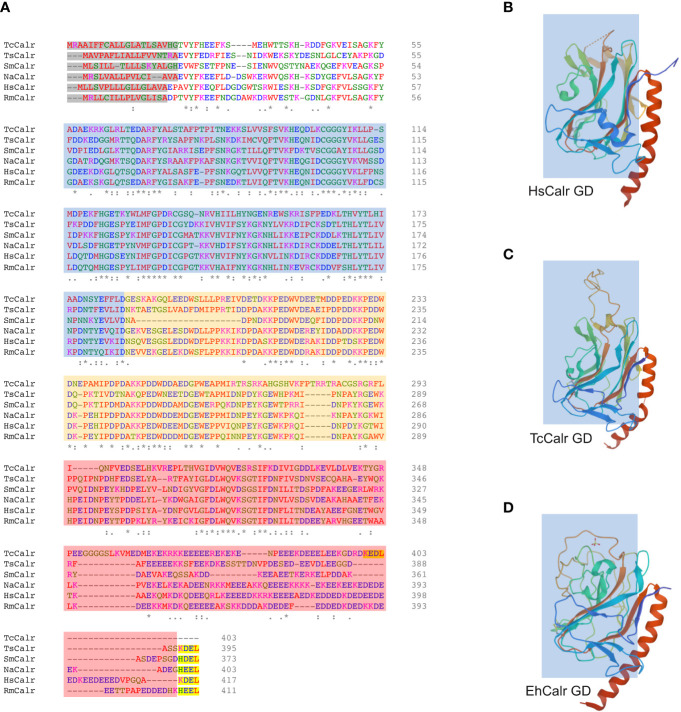
Alignment of different parasite Calr with HsCalr and crystal structure of the globular domain of TcCalr, EhCalr and Hs Calr. **(A)** Amino acid sequences were align using the ClustalOmega (1.2.4) tool. Signal peptide is highlighted in gray, the ER retention signal is in yellow. N, P, and C domains are shaded in blue, orange and red respectively. Genebank accession numbers: TcCalr: *Trypanosoma cruzi* calreticulin, AAD22175.1; TsCalr: *Taenia solium* calreticulin, AAK52725.1; SmCalr: *Schistosoma mansoni* calreticulin, AAA19024.1; NaCalr: *Necator americanus* calreticulin, CAA07254.1; HsCalr: *Homo sapiens* calreticulin, AAA51916.1; RmCalr: *Rhipicephalus microplus* calreticulin, AAR29940.1. An * (asterisk) indicates positions which have a single, fully conserved residue; a: (colon) indicates conservation between groups of amino acids with strongly similar properties; a . (period) indicates conservation between groups of weakly similar properties. **(B)** *Homo sapiens* recombinant globular domain, PDB ID: 3POS; X-ray diffraction 1.65 Å (30); **(C)** *Trypanosoma cruzi* strain CL Brener; recombinant globular domain, PDB ID: 5HCF; X-ray diffraction 2.15 Å (34); **(D)** *Entamoeba histolytica* strain HM-1:IMSS; recombinant globular domain in complex with glucose, PDB ID: 5HCA; X-ray diffraction 2.15 Å (34).

Calr is a hybrid protein containing ordered and disordered regions and does not have a complete and well characterized 3D structure. The N domain together with the N-terminal half of the C domain of HsCalr constitute a globular domain that is stable. The P and C-terminal tail of the C domain constitute 2 flexible arms (27, 41–43). A chimeric protein with the N domain and the N-terminal of the C domain connected by a linker sequence and lacking the P and the C-terminal of the C domain has been successfully crystallized (PDB ID: 3POS; 30). The crystal structure of MmCalr (PDB ID: 3O0W; 29), *Entamoeba histolytica* Calr (EhCalr) (PDB ID: 5HCA) and *Trypanosoma cruzi* Calr (TcCalr) (PDB ID: 5HCF; 34) have been obtained from similar constructs. Although the overall globular structure is very similar, comparison of the 3D structures of the globular domain of HuCalr with the protozoan Calr shows species differences at 10 sites (34). These differences could be exploited to block parasite Calr without affecting the host protein and could explain species-specific functional differences, while gaining insight into the conserved features and characteristics (**Figure 1**). The disordered nature of Calr results in a flexible protein that can acquire different conformations under physiological conditions depending on the cellular microenvironment (44).

## 3 Differential expression of calreticulin

Calr is not only differentially expressed in the life stages of parasites, but also through different developmental processes. Calr participates actively in infectivity. In addition to its ER location, TcCalr is present in other organelles and on the cell surface, mainly on the flagellum emergence area of *T. cruzi* trypomastigotes and promotes infectivity by stimulating host cell phagocytosis. (14, 45, 46). EhCalr is expressed in the phagocytic cup of *E. histolytica* suggesting a role in feeding and nutrition, as well as in intracellular vesicular structures and cell surface in trophozoites (47). Trophozoites over-express EhCalr during the early stages of the amoebic liver abscess, suggesting a potential role in adaptation to a new microenvironment and in *E. histolytica* invasiveness (48). In addition, EhCalr concentrates in the uropod, a structure at the ear of the amoeba that regulates cell motility, upon capping of surface receptors (47). A role in parasite feeding and transmission is also implied in the cattle tick *Rhipicephalus microplus* Calr (RmCalr) as it is present in the salivary glands acting as an anti-haemostatic, anti-inflammatory and immunomodulatory molecule (49).

Calr plays a role in defining the normal morphology of certain parasite species. The generation of *T. cruzi* epimastigotes which lacked an allele of the Calr gene or overexpressed said gene, resulted in parasites with morphological abnormalities, such as loss of the characteristic elongated form, alteration of the nuclear structure and effacement of the external nuclear membrane (50). Likewise, it has been shown that suppression of Calr expression, either through knockdown or iRNA-based techniques, reduces the virulence of certain parasites, such as *Radopholus similis* (51), as well as its capacity of surviving in a hostile intracellular environment; such is the case for *T. cruzi* (52).

The presence of Calr in reproductive organs of helminths implies its involvement in fertility and reproduction. In *Caenorhabditis elegans*, a free-living nematode, Calr is expressed in the different organs, including the sperm in the testis. Calr deletion mutants show reduced mating behavior and defects in sperm and oocyte development (53). High expression of Calr in the reproductive system is a common finding in helminths. Examples include *Opisthorchis viverrini* and *Schistosoma mansoni* (54, 55). In *Taenia solium*, TsCalr is differentially expressed during gametogenesis (56). It would be expected and suppression or neutralization of Calr expression in parasites would affect fertility and survival in the host. Indeed, oral immunization with TsCalr results in 37% reduction in worm burden and delayed development in a taenisosis experimental model. In addition, TsCalr is expressed in the tegumentary and muscle cytons, neutralization by specific anti-TsCalr IgA could interfere with nutrition across the tegument (17, 57). In *T. solium* organs involved in infection, such as the suckers, showed upregulation of Calr. Thus, the participation of parasite Calr in infectivity, transmission and nutrition is undeniable in protozoa, helminths, as well as in ectoparasites.

The importance of Calr during reproduction is maintained through evolution. Mammalian Calr participates in decidualization and embryo implantation in mice (58). Additionally, Calr is necessary for female fertility and ovarian function, is over-expressed in the perivitelline space upon egg activation, is involved in sperm-egg binding and in cell cycle resumption during fertilization (59, 60). Despite the different species-specific reproductive strategies among parasites, as well as mammals, the presence of Calr in the reproductive organs or as a means to promote invasiveness, highlights the significance of this multi-faceted protein for species survival.

## 4 Parasite calreticulin and the complement system

The complement system is a crucial humoral immune mechanism in the defense against pathogens, particularly bacteria, fungi and parasites. It is composed of three pathways: classical, alternative and the lectin pathway. Irrespective of their initial activation, the three pathways converge in the production of the complement protein C5 by the C5 convertase complex and the formation of the membrane attack complex (MAC), a multiprotein complex that becomes embedded in the plasma membrane of the invading microorganism and results in the formation of a pore that compromises membrane stability and induces cellular death through osmotic shock (61).

Despite their amino acid sequence differences, various forms of Calr secreted by different parasites share one function: they inhibit the initial steps of the classical complement pathway by binding to the collagenous domain of C1q (8, 62, 63), which, along with the serine proteases C1r and C1s, make up the C1 complex. Inhibition by Calr has been well studied in a *T. cruzi* model, it is independent of the calcium-binding capacity of Calr and rather pertains to the capacity of the pleiotropic protein to impede the binding of C1r-C1s to C1q, as well as to inhibit the cleavage of C4 by C1s (14). However, the TcCalr is not capable of displacing the proteases once they have formed a complex with C1q (14, 64). This is a key event favoring parasite survival, given that the classical pathway is interrupted in its earlier stages and this, in turn, impedes its capacity of producing C3b through the formation of the C3 convertase. C3b has two important actions: it acts as an opsonin by lodging itself in the outer layer of the plasma membrane of the pathogen, favoring its elimination by phagocytes, and it intervenes in the formation of the C5 convertase, which, as mentioned above, is essential for the integration of the MAC, the final effector mechanism of the proteolytic cascade (61). Indeed, *T. cruzi* trypomastigotes and epimastigotes which are deficient in TcCalr are more susceptible to complement-mediated elimination (52, 65–67), while the opposite —a greater expression of Calr confers resistance to killing by complement— also holds true (52, 68).

TcCalr has also been shown to inhibit the mannose-binding lectin (MBL) of the lectin complement pathway. MBL is analogous both in structure and function to C1q, but its preferential ligands are carbohydrate patterns expressed on the cell surface. Subsequently, bound MBL is recognized by MBL-associated serine protease (MASP) proteases and the lectin pathway is activated. Interestingly, TcCalr can also interact with L-ficolin (another important ligand participating in the lectin pathway), but, in this case, it does not seem to interfere with its capacity of binding to its natural ligand, such as lipoteichoic acid (LTA) and specific carbohydrate patterns. In the case of H-ficolin, TcCalr does not seem to interact with it, a fact that is likely explained by the low shared identity between L- and H-ficolins (69). Nevertheless, TcCalr is effective at inhibiting both classical and lectin complement pathways.

Other parasites whose Calr has displayed the capacity to inhibit the complement pathway include, *Trypanosoma carassii* (70), *E. histolytica* (16), *E. dispar* (71), *Brugia malayi* (72, 73), *Wuchereria bancrofti* (19), *Trichinella spiralis* (20, 74), *O. viverrini* (75), *Haemonchus contortus* (76, 77), *Necator americanus* (76, 78), *S. mansoni* and *S. japonicum* (79), among others. An apparent exception to this rule is found in the Calr of the tick *Amblyomma americanum*, which is present in the saliva of the specimen. It was thought that the presence of Calr in the saliva facilitated feeding by the tick through the inhibition of C1 and a purported antihemostatic function (14, 62, 80). Recent experimental data, however, did not show that *A. americanum* Calr is capable of inhibiting C1q, although it does bind to it (78). A recombinant form of Calr, which may have distinct functions relative to the native form, was used in this study, and this might explain the apparently contradictory result, but given the variety of functions that Calr serves, it would certainly not be unreasonable to think that Calr in the saliva has a role other than inhibiting complement.

*Triatoma infestans*, a hematophagous insect which acts as a vector for *T. cruzi*, also expresses Calr (81). During feeding, the insect secretes its Calr, TiCalr, into the host’s bloodstream, mediating beneficious effects, such as inhibition of complement pathway (82) and inactivation of clotting factors, including calcium. It is interesting that both the vector of Chagas disease and its causative agent secrete Calr in order to inhibit the complement system; this might be a coincidence or, more likely, an example of a coevolutive trait that has led *T. infestans* to be the principal vector of *T. cruzi*.

Another way in which parasite Calr is able to indirectly modulate the activation of complement is through binding of C-reactive protein (CRP), an acute phase reactant whose production is induced by IL-6 in the context of acute infection (83). CRP recognizes and binds multivalent ligands and then attaches to C1q, activating it. It has been shown that *H. contortus* Calr is capable of binding and inactivating CRP, thus preventing it from activating the classical complement pathway (83).

**Figure 2:**
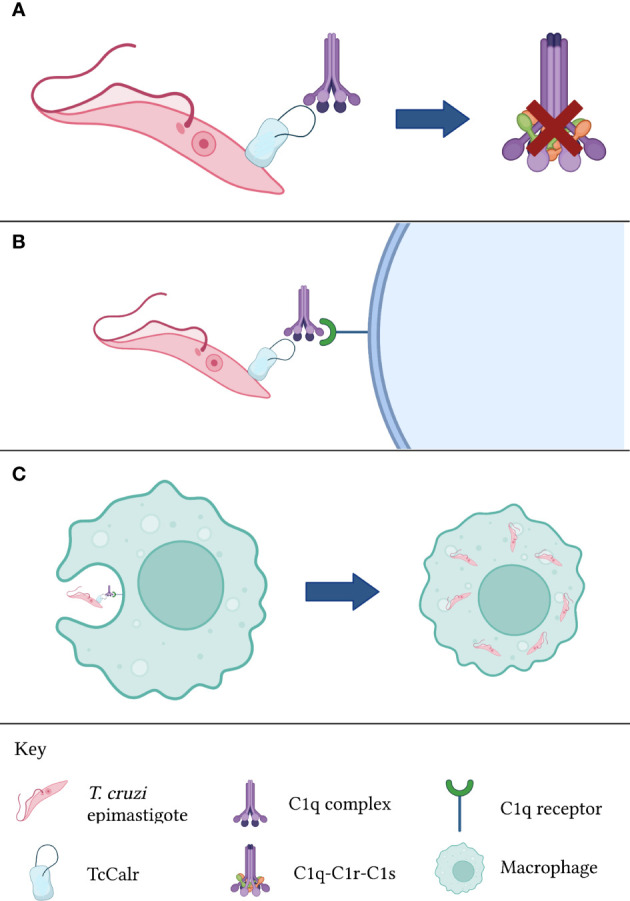
Calr from *T. cruzi* is inhibits complement activation. **(A)** TcCalr binds to C1q and impeding C1r and C1s proteolysis. **(B)** Once the TcCalr-C1q complex is formed, it is recognized by C1q receptors expressed on the surface of phagocytes. **(C)** Upon recognition, phagocytes engulf the epimastigote, promoting infectivity. TcCalr: *Trypanosoma cruzi* calreticulin.

In any case, the functions of parasite Calr regarding the complement system are not restricted to the inhibition of the latter. A possibly even more significant activity during parasitic invasions, is the increase in infectivity that Calr bound to C1q confers (10, 14, 64, 68, 84). Because C1q is recognized by a variety of surface receptors, including Calr itself (in this context, it will be referred to as cC1qR) and other complement receptors, C1q bound to Calr expressed on the surface of the parasite would promote its phagocytosis by macrophages and other cells belonging to the mononuclear phagocyte system (62), as well as cells in other types of tissues such as the placental syncytiotrophoblast (85) and endothelial cells (12). The relevance of Calr uptake by endothelial cells will be discussed in a subsequent section. In the specific case of macrophages, these cells, which are adept in eliminating intracellular pathogens, become dysfunctional when infected by *T. cruzi* trypomastigotes. For example, their antigen-presenting capacity to T cells becomes limited (86). Thus, infected macrophages act as reservoirs of the parasite, favoring a chronic infection. Indeed, these macrophages, which become almost constitutively activated, serve as a “means of transportation” for the parasite while also aiding in their infection of adjacent tissues through the release of extracellular vesicles containing infective *T. cruzi* trypomastigotes (87). The aforementioned process is illustrated in **Figure 2**. In regards to placental infection by *T. cruzi*, this is a mechanism that explains vertical transmission of Chagas disease, and it is aided by the recognition of C1q bound to parasite Calr by placental Calr (85). The great significance of the Calr-complement interaction is undisputed.

## 5 Calreticulin and cancer

An important function of the immune system is the so-called “immune surveillance”, whereby phagocytic cells constantly scout tissues in search for cells that exhibit changes compatible with malignant transformation, such as cells with hyperploidy, which overexpress Calr due to an unfolded protein response (UPR) (88). Indeed, Calr, in line with its myriad of physiologic actions in mammals, acts as a potent “eat me” signal that stimulates phagocytosis of, particularly, apoptotic cells and apoptotic remains, a phenomenon known as efferocytosis (89). As mentioned in the previous section, surface-expressed Calr is capable of binding C1q and MBL. These molecules act as opsonins, favoring the interaction between Calr and the phagocytic cell through two main mechanisms: binding of Calr to CD91 or to scavenger receptor A (SR-A) (89). This interaction allows for the uptake of the cell expressing Calr by the phagocyte. In healthy cells, the expression of “don’t eat me” signals, such as CD31 and CD47, counteract the “eat me” signals and negate efferocytosis (89, 90).

In the context of Chagas disease, an important antitumor role has been proposed for TcCalr. Epidemiological studies have shown a protective effect of *T. cruzi* infection against various types of cancers. Early experiments on this matter revealed that addition of trypomastigote extracts induced antitumor immunity in both animal and human models (81). *In vitro* studies have shown that TcCalr has antiangiogenic properties: it inhibits capillary growth and interferes with morphogenesis, proliferation and chemotaxis of endothelial cells (75, 81, 84, 91). Vasostatin, a fragment that lies between the N and P domains of Calr, mediates these effects (80). Further investigation on this topic has revealed that the exact region responsible for these antiangiogenic effects is made up of amino acid residues 131 to 159; it has been termed VC-TcCalr by the authors (92). VC-TcCalr has a strong dipole nature that allows it to interact with collagen-like tails and scavenger receptors, both of which are necessary to inhibit angiogenesis. As it stands, TcCalr can be internalized by endothelial cells through, at least, the two previously described mechanisms (CD91 and SR-A). Within the cell, Calr likely interacts with integrins (10) and other proteins involved in cellular signaling pathways (89) to modify processes pertaining to cellular adhesion, differentiation and proliferation. Of interest, abrogation of TcCalr uptake by fucoidan, a natural ligand of SR-A, inhibits the protein’s antitumor and antiangiogenic effects (12, 81, 91), implying two important notions: that SR-A is crucial in the endocytosis of TcCalr (and, likely, most forms of Calr) and that the antitumor effects of TcCalr are mediated largely in part by its capacity to disrupt endothelial architecture and capillary growth.

The explanation for this phenomenon is rather logical, albeit possibly reductionist. Malignant tumors are dependent on angiogenesis to satisfy their metabolic needs, which continue to increase following tumor growth. Proliferating cancer cells secrete a variety of endogenous angiogenic factors, including vascular endothelial growth factor (VEGF), basic fibroblast growth factor (bFGF), and transforming growth factor (TGF) α and β, which activate endothelial cells an initiate angiogenic processes (Nishida, 2006). If angiogenesis is deficient, as occurs in the presence of TcCalr, tumor cells suffer from starvation and become stressed. This chain of events initiates the autophagy and apoptosis programs, resulting in destruction of affected cells (93). During these events, externalization of Calr may also occur, stimulating efferocytosis (10, 12).

Another possibility is that Calr acts directly on cancer cells. A study in which various cancer cell lines were treated with TsCalr showed significant dose-dependent reductions in cell viability and tumor colony formation in cervix, ovarian, breast and colon cell lines. The antitumoral effects were also seen in cancer stem cells (11). Likewise, in an experiment conducted by Lu et al. (94), HepG2 (human hepatocarcinoma) cells were incubated with recombinant *Angiostrongylus cantonensis* Calr (rAcCalr). Results indicated that proliferation of HepG2 cells diminished and apoptosis was induced; the markers ATF6 and CHOP were distinctively elevated. Both ATF6 and CHOP participate in apoptosis induced by endoplasmic reticulum stress (ERS) (95). An important factor contributing to ERS is Ca^2+^ dyshomeostasis: the cation is important in post-translational processing, intervening in the folding and maturation of proteins (96). If Ca^2+^ within the ER is depleted, protein processing becomes impaired, which leads to the accumulation of unfolded or misfolded proteins within the ER. This, in turn, activates the UPR, in which ATF6 acts as a signaling protein, a response which attempts to correct the unfolded protein surplus, but usually leads to apoptosis (97). Given the Ca^2+^-binding properties of Calr, it is feasible that Calr induces changes in intracellular calcium concentrations within cancer cells, leading to the activation of the UPR and apoptosis.

The matter becomes even more complex when analyzing the interaction between the various Calr types. The ERS response, initiated either by starvation of the cancer cell or by a purported Ca^2+^-dependent mechanism, leads to the overexpression and externalization of ER-bound proteins, including HsCalr (98). As mentioned in the complement section, Calr itself can act as a receptor for C1q (cC1qR). Thus, HsCalr expressed by stressed tumor cells can bind to C1q and the complement protein can, in turn, bind to parasite Calr, the proposed initiator of the stress response. This protein complex can further interact with receptors expressed on the phagocyte surface (SR-A, CD91), acting as an opsonin (99). A fascinating Calr interplay is established, whereby parasite Calr and HsCalr interact, indirectly and directly, with each other, leading to the apoptosis and efferocytosis of cancer cells.

HsCalr and parasite Calr have an identity of about 50% (100, 101). As will be mentioned in a subsequent section, Calr is a highly immunogenic molecule. HsCalr exposed in response to certain chemotherapeutic agents used to treat cancer induces an immunogenic cell death (ICD), a process that entails processing of tumor antigens and the induction of a tumor-specific adaptive immune response (89). ICD is, in part, mediated by the aforementioned immunogenicity of HsCalr. Given the structural disparity between HsCalr and parasite Calr, the latter acts as a potent immunogen that favors processing of antigens derived from parasite Calr-bound cancer cells. Indeed, it has been shown that dendritic cells (DCs) that engulf tumor cells treated with recombinant TcCalr mature —represented by an increased expression of MHC II class molecules, CD80, and CD86, among other markers— in a dual canine tumor model (90). This maturation is likely to be followed by antigen presentation to both CD4+ and CD8+ lymphocytes through the antigen cross-presentation mechanism (81), leading to an adaptive immune anti-tumor response.

## 6 Immunomodulatory effects of parasite calreticulin

The production of excretion/secretion (E/S) products by parasites has been long recognized as a crucial event in host invasion and parasite survival, as well as in the establishment of the parasite-host relationship. A large number of parasites produce Calr as part of their E/S products. Exoproteome analysis of different parasites, protozoa and helminths, predicts that Calr is secreted by the classical pathway due to the presence of a signal peptide in the N-terminal of the protein (102, 103). Parasites in culture have also been shown to secrete Calr, some examples include *T. cruzi*, *H. contortus* and *T. solium* (77, 84, 104). Moreover, extracellular vesicles have recently been considered as important vehicles for several E/S products and research is rapidly expanding (105, 106). Calr has been shown in extracellular vesicles from trophozoites of *E. histolytica* and *T. cruzi* (107–109), as well as in the extracellular vesicles produced by helminths (110, 111). This implies that parasite Calr released in extracellular vesicles may engage in the host-parasite interplay as mediator of immune evasion strategies and modulator of the immune response. In this section, the intention is to illustrate the vast array of functions that Calr fulfills in its role as an E/S product, mainly in terms of immune regulation and modulation.

Calr seems to be a crucial component of the parasites’ invasion molecules repertoire. As mentioned before, the capacity of Calr of numerous parasite species to bind to C1q and thus allow uptake by macrophages and other phagocytes is a key event mediating infection. Moreover, the capacity of *T. cruzi* epimastigotes to penetrate human host cells diminishes when the host expression of surface Calr is deficient (112), further consolidating the concept of the Calr interplay in various forms of parasitism. Calr is also capable of binding to other molecules. For example, TcCalr binds to thrombospondin 1 (TSP-1) expressed on the surface of embryo fibroblasts and enhances trypomastigote infection of these cells (113). It is also noteworthy that Calr is expressed in regions pertaining to infectivity, such as the suckers and scoleces of *Echinococcus granulosus* and *T. solium*, two platyhelminthes that use these structures to adhere itself to the intestinal mucosa (56, 114). Furthermore, Calr could also regulate the excretion of other E/S products through its role as an ER-resident chaperone. *Leishmania donovani* promastigotes transfected with the P domain of *L. donovani* Calr had a marked reduction in E/S product formation, as well as a shorter survival of these specimens within macrophages (115).

In addition to its invasive properties, Calr has demonstrated a capacity to modify immune responses. The Calr of the root-knot nematode *Meloidogyne incognita* suppresses PAMP-triggered immunity (PTI) whilst infecting its host (116); PTI can be considered the innate immune response of plants. A similar effect was encountered when studying the function of *Heterodera avenae* Calr (HaCalr) (117); the same study showed that treatment of plants with HaCalr reduced the intensity of the respiratory burst and facilitated *H. avenae* parasitism in early stages. Insects fall victim to parasitism as well: an important immunoprotective mechanism against parasites is the encapsulation of the invading organism, a process that requires hemocyte spreading to occur. The Calr of *Cotesia rubecula* (118) and *Pteromalus puparum* (119) inhibit hemocyte spreading, thus thwarting encapsulation and promoting parasitism. *Radolpholus similis*, a migratory plant nematode, secretes a Calr that serves many functions, such as limiting the immune response of the host (which aids in the infection process), stimulating differentiation of parasite cells, reproduction and obtaining nutrients, as well as reproduction and establishing a host-parasite relationship (51).

In light of its multiple effects not only on the host immune response, but on parasite development and survival, it is only logical to assume that Calr plays a significant role in inducing immune responses. Indeed, Calr is a potent immunogen, a property which will be further discussed in the following section regarding vaccines. A particularly interesting property of the Calr of numerous parasites is its capacity to polarize the host’s immune response. Upon interaction with a specific antigen, naïve T CD4+ cells can differentiate into various functionally distinct subtypes, the principal three being Th1, Th2 and Th17. Interleukin (IL)-12 favors Th1 polarization; this subtype produces IFN-γ, a cytokine which intervenes in the defense against intracellular microorganisms (e.g., bacteria, protozoans). The Th2 subtype is induced by IL-4: it is the main subtype associated with the response against nematodes and platyhelminthes; it also participates in allergic reactions (120). Finally, Th17 polarization requires the presence of pro-inflammatory cytokines, such as IL-1β and IL-6, in addition to TGF-β (121). Once primed, this subtype secretes IL-17A, a chemokine that recruits neutrophils and participates in defense against extracellular pathogens. Calr is able to influence the polarization of the T cells, mainly to the Th1 and Th2 subtypes.

**Figure 3:**
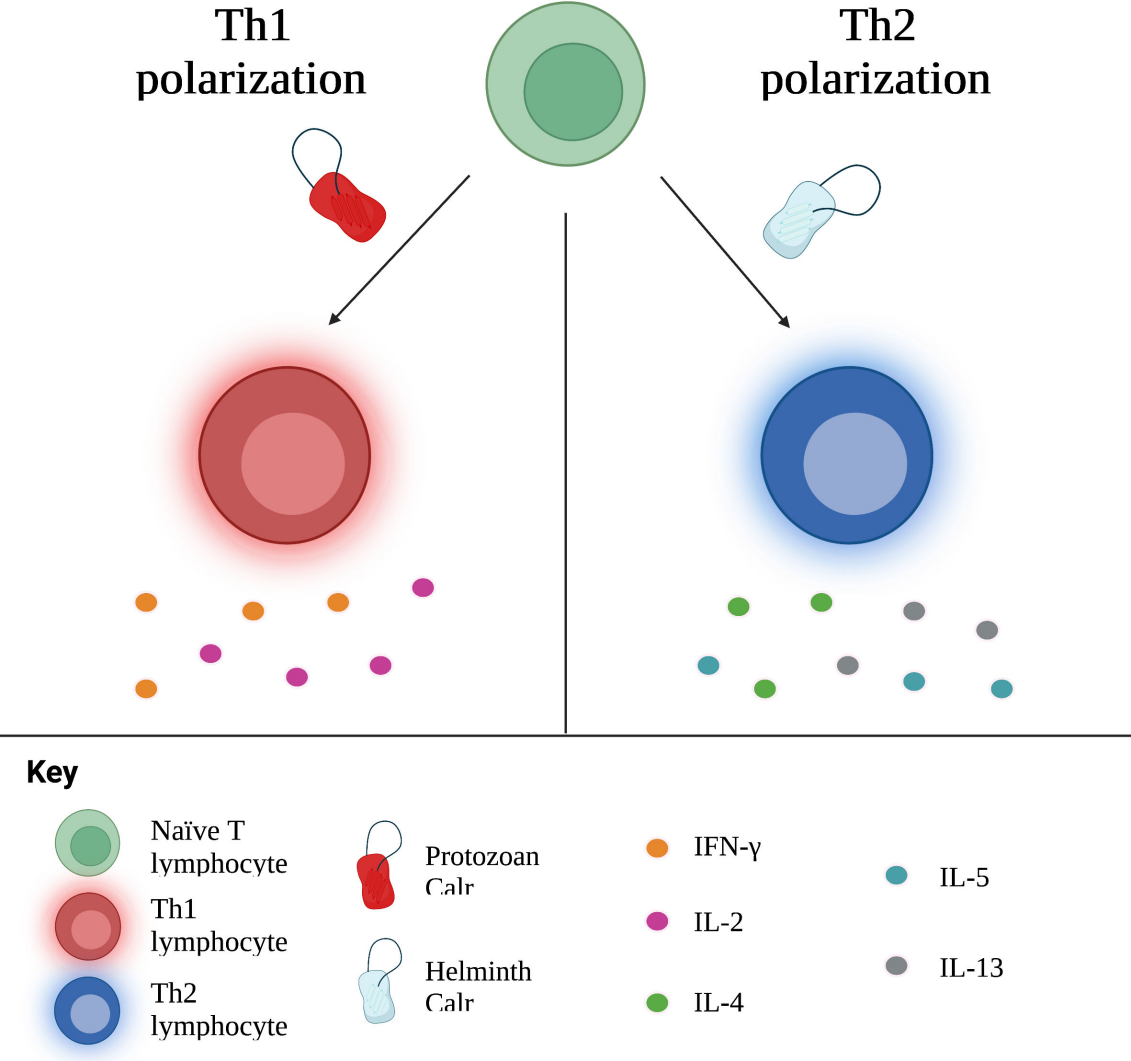
Calr influences the polarization of naïve T cells towards Th1 or Th2 subtypes, with concurrent production of associated cytokines. In general, Calr derived from protozoans induces a Th1 response, whereas helminth Calr favors a Th2 response. Th, T helper; IFN, interferon; IL, interleukin.

Theoretically, protozoans such as *T. cruzi* and *L. donovani*, which are intracellular pathogens, should prompt a Th1 response, while multicellular parasites elicit Th2 responses. Of note, administration of Calr of these types of parasites generally prompts the same immune response as the parasite it belongs to. In other words, protozoan Calr induces a Th1 response; nematode and platyhelminth Calr, a Th2 response. *E. histolytica*, an amoeba responsible for hepatic abscess in humans, triggers the release of pro-inflammatory cytokines, including IFN-γ, related to Th1, as well as TGF-β1 and IL-17, associated with Th17 (16, 48). Furthering this idea, amongst proteins secreted by *L. donovani*, another protozoan, is Calr, identified as a stimulator of the Th1 response (122). On the other hand, various studies involving hamster immunization with recombinant *T. solium* Calr (rTsCalr) showed a potent induction of Th2-response related effects, including: production of IL-4, IL-5, IL-10 and IL-13; hyperplasia of goblet cells; and increased mucus synthesis (5, 17, 104). Likewise, the nematode *Heligmosomoides ploygyrus* is known to skew the immune response towards a Th2 phenotype. In the study conducted by Rzepecka et al. (18), *H. polygyrus* Calr stimulated IL-4 and IL-10 synthesis by T CD4+ lymphocytes isolated from mice infected by *H. polygyrus*, suggesting a Th2 polarization. Nevertheless, certain models of infection do not demonstrate such a straightforward relationship between Calr from a particular type of parasite and cellular polarization. Recombinant *S. japonicum* Calr (rSjCalr) should, purportedly, initiate a Th2 response, given that *Schistosoma* spp. are platyhelminthes. In experimental studies, however, incubation of mice splenocytes and T CD4+ cells with rSjCalr resulted in elevation of IFN-γ and TNF-α, both cytokines involved in the Th1 response (21, 81). Interestingly, mild increases in IL-4 were also detected in this model; similar results were found in the amoebic liver abscess model by González-Rivas et al. (16), in which Th2 cytokines, including IL-5, IL-10 and TGF-β1, were upregulated in the acute phase of infection. This suggests that Calr is pleiotropic in its immunomodulatory properties, and that some of the differences encountered among the Calr from different parasites could, at least in part, be responsible for the different polarization cues elicited, as shown in **Figure 3**. In addition, since parasites undergo different stages upon infection of the host, other molecules differentially excreted/secreted could modify the resulting profile, a process that is time-dependent. Irrespective of its specific role in the tailoring of the immune response, it is undeniable that this molecule is key in establishing the phenotype of specific immune responses, although the involvement of additional modulating factors are likely to play a role in most, if not all, settings (104).

How does Calr induce such significant changes in the immune response against the parasite that secreted it? Various possibilities exist. First, Calr itself could directly modify signaling pathways involved in immune activation, maturation and regulation through two (or, likely, more) mechanisms: Ca^2+^ binding and interaction with signaling proteins (123). Recombinant *N. americanus* Calr has been shown to bind to the cytoplasmic domains of αIIb and α5 integrins independently of Ca^2+^ (124). This interaction is of importance, given the abundance of integrin motifs involved in immune regulation and many other aspects of cell function. In some cases, Calr does not influence polarization, but rather suppresses T cell activity altogether. The E/S products of *H. contortus*, including Calr, inhibit T cell proliferation and promote apoptosis by reducing IL-2, IL-4 and IFN-γ expression and increasing that of IL-10 and TGF-β (125). The latter two cytokines are characteristic of immunoregulatory responses, and they are chiefly produced by T regulatory lymphocytes (Tregs). The induction of a regulatory phenotype during prolonged parasitic infections, particularly when helminths are involved, is a well-known outcome of these processes. Indeed, helminths are considered “master regulators”, with properties that allow for long term chronic infections.

It has been proposed that parasites play a substantial role in the “education and fine-tuning” of the immune system. In the absence of stimuli that favor the modified Th2 response, other effector responses, including the Th1, Th17 and allergy-related Th2 phenotypes, may become rampant. Bacteria and viruses are also important in training the immune system (126). The hygiene hypothesis attempts to provide an explanation of the recent increase in inflammatory and allergic conditions worldwide, with a higher incidence in developed countries (127). This hypothesis suggests that lack of exposure to pathogens during infancy (a period where significant immune maturation occurs) is, in part, a causative factor of the rise in conditions whose main feature is immune dysregulation (126). Furthermore, the “old friends” hypothesis states that immune competence is developed in the presence of organisms with whom mammals hold an extensive coevolutionary background (128). In similar fashion as the hygiene hypothesis, the “old friends” hypothesis proposes that diminished exposure to microorganisms and parasites in industrialized nations is a purported mechanism for the development of a hyperactive immune response by the population of these territories. Parasites seem to be of particular importance in the immune education, not only through the induction of modified responses and Tregs, but also by way of secretion of immunomodulatory products, including Calr (2). Owing to its capability to influence the polarization of the immune response of the host, Calr is likely an important mediator in the process of immune regulation. This protein has persisted throughout the spectrum of eukaryotic organisms; thus, from a coevolutionary perspective, its participation in immune education and in the host-parasite relationship cannot be understated.

## 7 Calreticulin-based parasite vaccines

In spite of the coincidence in amino acid sequence and function between parasite and host Calr, the former is highly immunogenic and, as such, has been used as an immunogen in order to generate immunity against various infectious parasites. Yadav et al. (19) inoculated mice with recombinant Calr from *B. malayi* (rBmCalr), the causative agent of lymphatic filariasis (also known as elephantiasis). It was shown that rBmCalr activated peritoneal macrophages and splenocytes stimulated the production of cytokines characteristic of the Th1 response, such as IL-6, IL-2, IFN-γ and TNF-α. There were also elevated titers of antigen-specific IgG1, IgG2a, IgG2b and IgG3. Moreover, mice immunized with rBmCalr had lower worm burden in comparison with the control group, in account of the immune effects of vaccination. In another study, Winter et al. (129) attempted to immunize mice against *N. americanus* through the administration of this parasite’s Calr (NaCalr). In the context of *N. americanus* infection, the habitual response is Th2, with high IgE titers. Indeed, mice inoculated with NaCalr exhibited increased titers of IgG1, which is produced in a Th2-dependent manner (130). Interestingly, high levels of IgG1 did not necessarily correlate with protection against parasite challenge; this depended on the form of NaCalr administered. Free NaCalr reduced the worm burden, albeit it did not significantly raise IgG1 titers; on the contrary, encapsulated NaCalr increased IgG1 concentrations, but this increase was not met with a corresponding reduction in parasite burden. This information is relevant in two ways: it shows that mere administration of Calr is not sufficient to generate adequate immunity, rather its “presentation” must also be taken into account; and that the generation of an “adequate” immune response may not represent the only mechanism by which infection is eliminated.

In general, a strong humoral response against Calr is elicited upon immunization, and this response may or may not be significant in terms of protection against and elimination of infection, as aforementioned. Bossard et al. (15) showed that inoculation of recombinant *Trypanosoma congolense* Calr stimulated IgG1 and IgG2 production in mice and was associated with a more extended survival when compared with the control group. Similar results were found by el Gengehi et al. (131) upon immunization of mice with radiation-attenuated cercariae of *S. mansoni*. On the other hand, Ramírez-Toloza et al. (132) reported that immunization with recombinant *T. cruzi* Calr had the opposite effect: it promoted infectivity. As mentioned in a previous section, the binding of antibodies to the exposed Calr promotes infectivity of *T. cruzi* through an antibody receptor-mediated mechanism, whereby phagocytes recognize the Fc region of the bound antibody and this mediates the uptake of the parasite. Thus, the parasite’s mechanisms of virulence and infectivity must also be taken into account in the design of an effective vaccine.

Parasite Calr is phylogenetically very distant from the human form of the protein. This significantly reduces the probability of generating autoreactive antibodies against HsCalr following immunization with parasite Calr (15). However, the possibility of cross reactivity between phylogenetically close species is possible. The humoral response targeting the Calr of a specific parasite species could target the Calr of a phylogenetically related species. Indeed, Parizi et al. (133) based their study on this rationale. They administered recombinant Calr of one of two related blood-sucking ticks, *R. microplus* (rRmCalr) and *Haemaphysalis longicornis* (rHlCalr) to bovines. The serum extracted from a cow inoculated with rRmCalr reacted against both rRmCalr and rHlCalr, and vice versa. Another important finding of this study is that the sera of vaccinated bovines also recognized the native form of either protein, demonstrating that, at least in the case of *R. microplus* and *H. longicornis*, the recombinant form of Calr can be used instead of the native form to generate immunity with similar efficacy. Similarly, Kumar et al. (134) observed variable degrees of cross-reactivity against Calr derived from *R. microplus* and *Hyalomma anatolicu*m, with protective functions in both cases. In another study, researchers fed *R. microplus* females with serum enriched with antibodies directed against rRmCalr. Their findings showed that no significant changes in terms of tick viability and infections occurred (135), suggesting that direct inhibition of Calr, rather than the generation of a structured immune response, is insufficient to limit parasitemia.

Besides its capacity to induce a humoral response, vaccination with Calr favors polarization of T CD4+ cells, usually to the type of response associated with the parasite from which the Calr derives, as previously described. A cellular effector response by other immune cells may also be seen. The syrian hamster *Mesocricetus auratus* can withstand *T. solium* infection and also allows the helminth to develop, albeit without reaching gravidity. In this model, inoculation of rTsCalr resulted in an increased production of IgA and IgG, goblet cell hyperplasia and overexpression of IL-4 and IFN-γ; importantly, these responses were associated with a reduction in parasite burden (17). Production of IL-4, IL-5 and IL-10, as well as proliferation of spleen and mesenteric lymph nodes cells, was shown to increase upon *in vitro* stimulation with rTsCalr and *T. solium* extract (104). Likewise, Salazar et al. (136) demonstrated a reduction in genotoxicity following oral vaccination of hamsters with rTsCalr. Genotoxicity is an indirect measurement of tapeworm burden, because these organisms are capable of damaging DNA through both immune and non-immune mechanisms. Thus, a reduction in tapeworm burden was achieved after use of rTsCalr. Finally, vaccination with rTsCalr may have other beneficial effects other than limiting parasitemia. An inverse correlation has been established between inflammatory diseases and helminth infection (137). Certain species of parasites secrete various excretion/secretion products, Calr among them, that attenuate the inflammatory response in order to create a favorable environment. In an murine experimental model of trinitrobenzenesulfonic acid (TNBS)-induced colitis, administration of rTsCalr increased the production of IL-4, IL-10, IL-13 and TGF-β; moreover, it reduced findings associated with colitis, such as weight loss, intestinal bleeding, genotoxicity and microscopic inflammation (5). This highlights the therapeutic potential of parasite Calr in the setting of inflammatory diseases.

## 8 Diagnostic uses of parasite calreticulin

Calr has also garnered attention as a means to diagnose certain parasitic infections in both human and non-human hosts. Chen et al. (138) found an abundance of Calr in the E/S products of the nematode *Angiostrongylus cantonensis* in the setting of eosinophilic meningitis. The detection of Calr and other immunoreactive proteins using immune techniques could be helpful in the diagnosis of this condition. In a similar manner, Calr was detected in two stages of development of another nematode, *Trichinella britovi*: muscle larvae and adult worms. Given this commonality, it is likely that Calr will be useful in the early detection of infection by *T. britovi* in endemic regions (139). An additional study performed predictions of the B-cell epitopes of TsCalr, which could potentially bind to antibodies and act as a serum biomarker for the diagnosis of neurocysticercosis (140). Furthermore, a recombinant form of *Acanthamoeba castellanii* Calr, the etiologic agent of granulomatous amebic encephalitis (GAE), was recognized by the sera of rats infected with GAE, highlighting its diagnostic capabilities (141).

Despite its purported advantages as a diagnostic tool, Calr could produce false positives in specific contexts. In an attempt to diagnose schistosomiasis caused by *S. mansoni*, a parasite which expresses Calr in cercariae and adult stages (142), researchers found high levels of cross-reactivity in patients infected by *E. histolytica* and *Ascaris* spp. Particularly, the N- and P-domains of Calr showed less sensitivity than the C-domain, likely owing to the less conserved structure of the latter amongst parasites (143). A decrease in sensitivity has also been shown when using an ELISA targeted to TcCalr in sera of patients positive for the protozoa *Leishmania mexicana*, *L. donovani*, *T. rangeli* and *Crithidia fasciculata* (144). Moreover, Morgan et al. (145) conducted a proteomic analysis of *Sarcoptes scabiei* in which they showed that Calr from the acarus had various homologs from other related mites and ticks, rendering its diagnostic utility almost null. In other species of parasites, such as *Leishmania infantum*, Calr was not immunogenic enough to act as an adequate serodiagnostic marker (146).

The above discussion suggests that Calr is not a sufficiently precise diagnostic marker, at least when employing immunological techniques for its detection. This is largely due to the cross-reactivity between Calr of different parasites. An interesting prospect is included in the study by Santucciu et al. (147), who amplified a specific Calr sequence in order to characterize different varieties of *E. granulosus*. Following this idea, it could be possible to build a repertoire of sequences of Calr belonging to various species of parasites in order to increase the sensitivity and specificity of Calr-based diagnosis, particularly through the use of the polymerase chain reaction (PCR) technique.

## 9 Calreticulin in wound healing and autoimmunity

Calr is important in the wound healing process. rTcCalr has been shown to be more effective than HsCalr in this setting, requiring lower concentrations to promote proliferation of fibroblasts, migration of epithelial cells and formation of granulation tissue (13, 148). Interestingly, these effects were only observed when the whole protein was administered, suggesting that the protein in its entirety or a large fragment of it is necessary to promote re-epitelization (148).

A role for both parasite Calr and HsCalr has been proposed in the context of autoimmune disorders, although much research is needed in this field. For instance, Ribeiro et al. (149) found that immunization of mice with rTcCalr in conjunction with Freund’s adjuvant induced a humoral response that resulted in immunopathological changes associated with chronic forms of Chagas’ disease, such as degenerative changes of cardiac muscle and infiltration of this tissue by mononuclear cells. Whether these changes are mediated by autoantibodies or by unknown pathological mechanisms is currently not known, but a causative role for Calr is clear. HsCalr has been linked to various autoimmune diseases, such as rheumatoid arthritis, systemic lupus erythematosus and other rheumatic diseases (63, 150). Interspecies conserved epitopes in protozoan proteins have been shown to be poorly immunogenic; thus, do not represent efficient B-cell epitopes (151). If the conserved epitopes among phylogenetically diverse Calr intervene in the pathogenesis of autoimmunity, this remains to be seen. Nevertheless, the use parasite Calr as immunomodulators is still a plausible strategy.

## 10 Conclusions

Calr is a pleiotropic molecule that is highly conserved amongst eukaryotes, owing to its myriad of functions in diverse cellular and molecular contexts. In the setting of parasite biology, Calr develops various roles, which vary depending on the particular species: it inhibits the classical complement pathway activation by binding to C1q and preventing C1r and C1s activation; it intervenes in the recognition of the parasite by phagocytes and aids in its infectivity, to the extent that Calr-deficient *T. cruzi* epimastigotes demonstrate poor infectious capabilities, as well as a reduced intracellular survival; and it modulates several other immune mechanisms of the host with which it interacts. These immunomodulatory properties are precisely what make Calr a prospective molecule in basic and clinical research, given that, likely, many of its functions remain to be elucidated. Its capacity to decrease the viability of different tumor cells is of particular interest in regards to cancer biology and treatment. Finally, the “polarizing” properties of Calr in terms of immunity, particularly in that it can favor Th2 responses, make it a promising candidate in the management of inflammatory and, possibly, autoimmune conditions in a prophylactic manner, as aforementioned. Truly, when it comes to Calr, the possibilities are nearly endless.

## Author contributions

FM and DE generated the idea for the review and wrote the manuscript. FM and DE designed the figures and DE elaborated the table. FM and AF read and corrected the manuscript. All authors contributed to the article and approved the submitted version.
